# Supplementary material for: TUBG1 missense variants underlying cortical malformations disrupt neuronal locomotion and microtubule dynamics but not neurogenesis
Source: Nat Commun. 2019 May 13;10:2129. doi: 10.1038/s41467-019-10081-8 (PMC6513894; doi:10.1038/s41467-019-10081-8)
Supplement: Supplementary file 1 — Supplementary Information [file 41467_2019_10081_MOESM1_ESM.pdf]

Supplementary Information

TUBG1 missense variants underlying cortical malformations disrupt neuronal locomotion and microtubule dynamics but not neurogenesis

E. L. Ivanova et al.

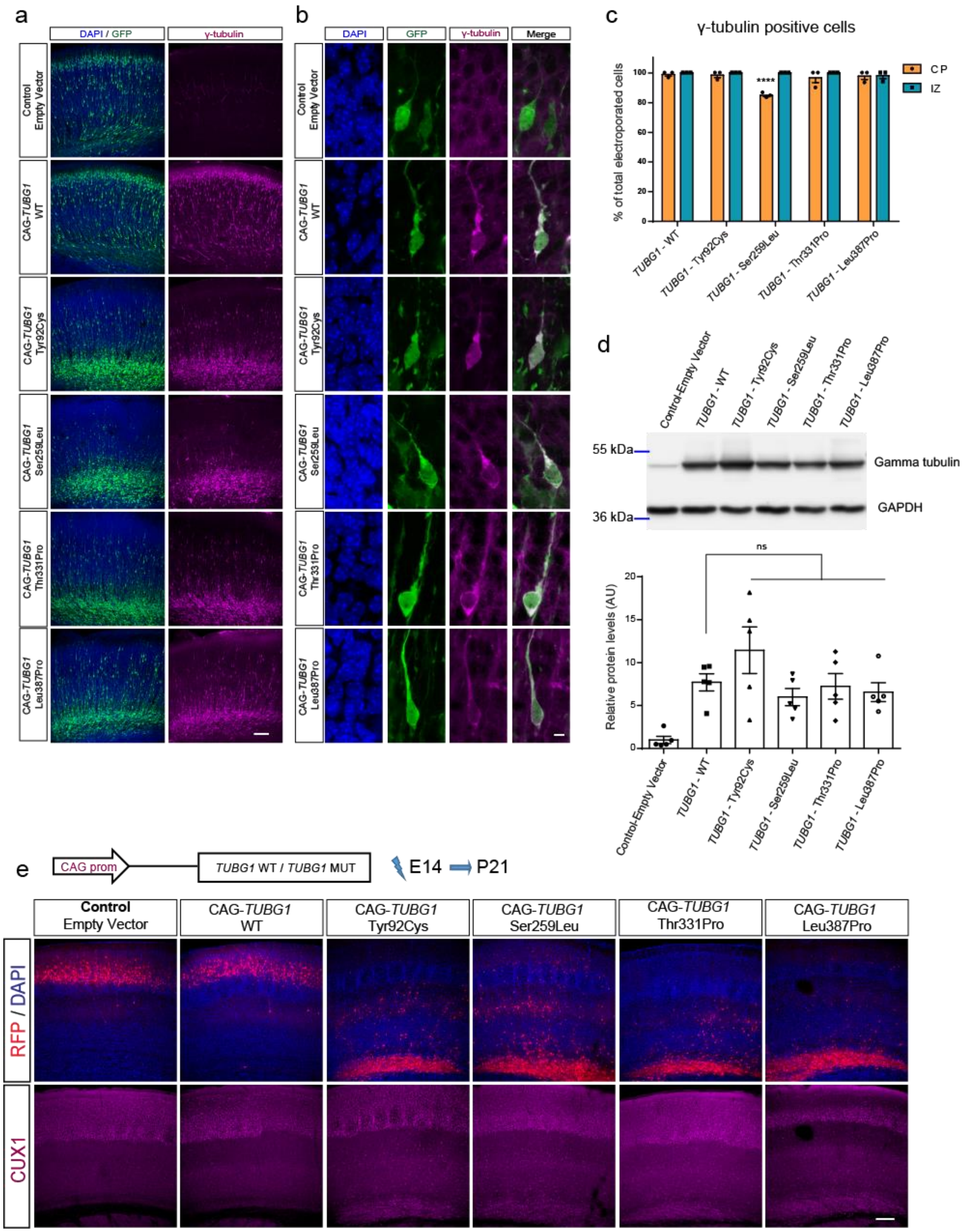

Supplementary Figure 1. TUBG1 pathogenic variants overexpression. (**a-b**). TUBG1 overexpression in electroporated cortical neurons. Coronal sections of E18.5 brains electroporated at E14.5 with either a control-empty vector, the CAG driven WT form or one of the four *TUBG1* mutated forms

and co-electroporated with a GFP-encoding reporter (green). (**a**) Sections were stained for gamma-tubulin (magenta) and counterstained with DAPI (blue). Scale bar 150µm. (**b**) Magnification of (**a**) showing an electroporated cell, scale bar 5µm. (**c**) Percentage of electroporated cells (GFP^+) overexpressing gamma-tubulin; n=3 embryos per condition. Two-way ANOVA, **** $p < 0.0001$ compared to WT. (**d**) Gamma-tubulin levels in Neuro2a cells transfected respectively with: a control-empty vector, TUBG1-WT, TUBG1-Tyr92Cys, TUBG1-Ser259Leu, TUBG1-Thr331Pro, TUBG1-Leu387P. Upper panel: Gamma tubulin detected by immunoblotting; lower panel: densitometry quantitation of immunoblots from n = 5 independent transfections, One way ANOVA with Dunnett's multiple comparisons test, ns: not significant vs WT. (**e**) Coronal sections of P21 brains electroporated at E14.5 with either a control empty vector, the CAG driven WT form or one of the four TUBG1 mutated forms and co-electroporated with pCAG-mScarlet (red). Sections were stained for the upper-layer marker Cux1 (magenta) and counterstained with DAPI (blue). Scale bar 200µm. Histograms represent mean ± s.e.m. Source data are provided in the Source Data file.

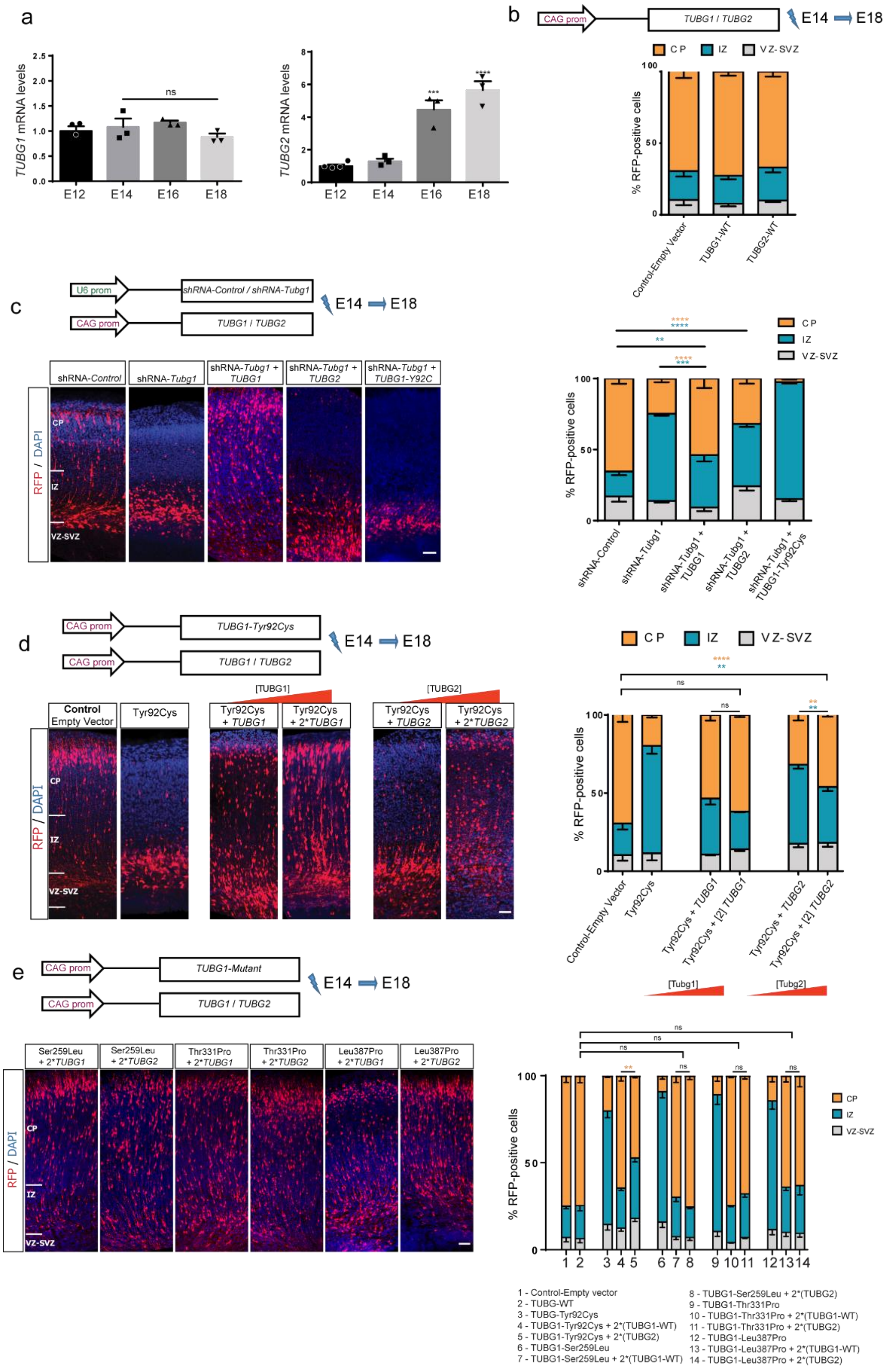

Supplementary Figure 2. TUBG1/TUBG2 during cortical development.

(**a**) Tubg1 (left panel) and Tubg2 (right panel) mRNA levels in mouse cortices at different developmental stages (E12, E14, E16, E18), n=3 independent samples, normalized to GAPDH and relative to levels at E12.5, One way ANOVA with Dunnett's multiple comparisons test, *** $p < 0.001$, **** $p < 0.0001$. (**b**) Percentage of electroporated cells in 3 cortical regions: CP, IZ, VZ/SVZ at E18.5 after electroporation at E14.5 with respectively: Control-empty vector, TUBG1 or TUBG2. Data are from 3 embryos per condition, one way ANOVA with Dunnett's multiple comparisons test. (**c**) Left: coronal sections of E18.5 brains electroporated at E14.5 with respectively: control sh-RNA, Tubg1sh-RNA, or Tubg1-sh-RNA together with either TUBG1, TUBG2 or TUBG1-Tyr92Cys and a RFP reporter (Red), stained with DAPI (blue). Scale bar 50 µm. Right: Percentage electroporated cells in 3 cortical regions: CP, IZ, VZ/SVZ. Data from at least 3 embryos per condition were analyzed with Two-way ANOVA with Tukey's multiple comparisons test ** $p < 0.01$, *** $p < 0.001$, **** $p < 0.0001$. (**d**) Left: coronal sections of E18.5 brains electroporated at E14.5 with respectively: Control-empty vector, TUBG1-Tyr92Cys, TUBG1-Tyr92Cys together with TUBG1 or TUBG2 (Rescue), stained with DAPI (blue). For rescue experiments, two different concentration ratios TUBG1/TUBG2:TUBG1-Tyr92Cys were tested: 1:1 and 2:1. Scale bar 50µm. Right: Percentage fluorescent cells in 3 regions: CP, IZ, VZ/SVZ. Data from at least 3 embryos per condition were analyzed with Two-way ANOVA with Tukey's multiple comparisons test; ** $p < 0.01$, **** $p < 0.0001$. (**e**) Left: coronal sections of E18.5 brains electroporated at E14.5 with respectively: TUBG1-Ser259Leu, TUBG1-Thr331Pro, TUBG1-Leu387Pro, and each TUBG1 mutant form together with TUBG1 or TUBG2 (Rescue). For rescue experiments, the ratio WT:mutant was 2:1. Sections were stained with DAPI (blue). Scale bar 50µm. Right panel: Percentage fluorescent cells in 3 regions: CP, IZ, VZ/SVZ, n=3 embryos per condition. Two-way ANOVA with Tukey's multiple comparisons test, ** $p < 0.01$.

CP, cortical plate; IZ, intermediate zone; VZ/SVZ, ventricular zone/subventricular zone. Histograms represent mean ± s.e.m. Source data are provided in the Source Data file.

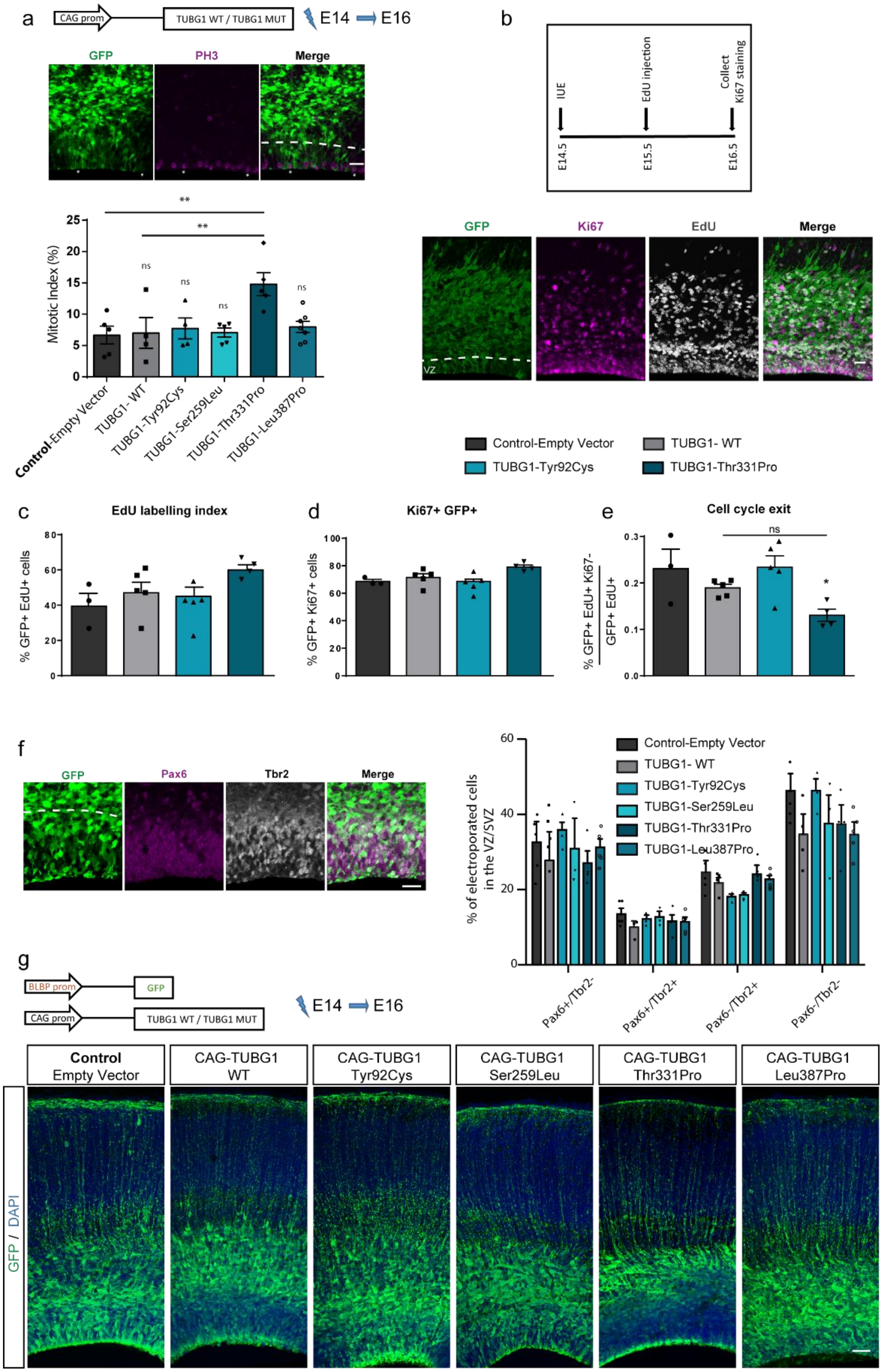

Supplementary Figure 3. Effects of TUBG1 variants on neural progenitors.

(**a**) Mitotic index study. Upper panel: E16.5 cortices, electroporated at E14.5 with WT or mutant TUBG1 and a GFP reporter (green), immunolabeled for the mitotic marker PH3 (magenta). Dotted line delimits the VZ for quantifications, scale bar 30µm. Lower panel: Percentage double-positive $GFP^{+}PH3^{+}$ cells reported to the total number of electroporated cells (mitotic index). Data from at least 4 embryos per condition were analyzed with One-way ANOVA with Dunnett's post hoc test, compared to WT or control-empty vector; ** $p < 0.01$. (b-e) Cell cycle exit assay. (**b**) Upper panel: schematics of the experimental design. Embryos were *in utero* electroporated at E14.5, received EdU injection at E15.5 and were analyzed at E16.5. Lower panel: E16.5 brain sections with electroporated cells in green (GFP), immunolabeled for the proliferative marker Ki67 (magenta) and EdU (grey), dotted line delimits the VZ for quantifications, scale bar 30µm. (c-e) Percentage electroporated cells having incorporated the EdU ($GFP^{+}EdU^{+}$) (**c**); percentage electroporated cells engaged in the cell cycle ($GFP^{+}Ki67^{+}$) (**d**); percentage electroporated cells that have exited the cell cycle ($GFP^{+}EdU^{+}Ki67^{-}/GFP^{+}EdU^{+}$, cell cycle exit index) (**e**). Data from at least 3 embryos per condition were analyzed with One-way ANOVA with Dunnett's post hoc test, compared to WT or control-empty vector. (**f**) Cell fate assay. Left: E16.5 cortices, electroporated at E14.5 with WT or mutant TUBG1 and a GFP reporter (green), immunolabeled for the apical progenitor marker Pax6 (magenta) and the basal progenitor marker Tbr2 (gray). Dotted line delimits the VZ/SVZ for quantifications. Scale bar 30µm. Right: Relative percentages of apical progenitors (Pax6+Trb2-), new-born intermediate progenitors ($Pax6^{+}Tbr2^{+}$), basal progenitors ($Pax6^{-}Tbr2^{+}$) and new-born neurons ($Pax6^{-}Tbr2^{-}$). Data from at least 3 embryos per condition were analyzed with One-way ANOVA with Tukey's post-hoc test. (**g**) E16.5 cortices co-electroporated at E14.5 with WT or mutant TUBG1 together with GFP (green) under the control of the apical progenitor promoter BLBP, counterstained with DAPI (blue), allowing the visualization of radial glial fibers. Scale bar 50µm. VZ: ventricular zone; SVZ: subventricular zone. Histograms represent mean ± s.e.m. Source data are provided in the Source Data file.

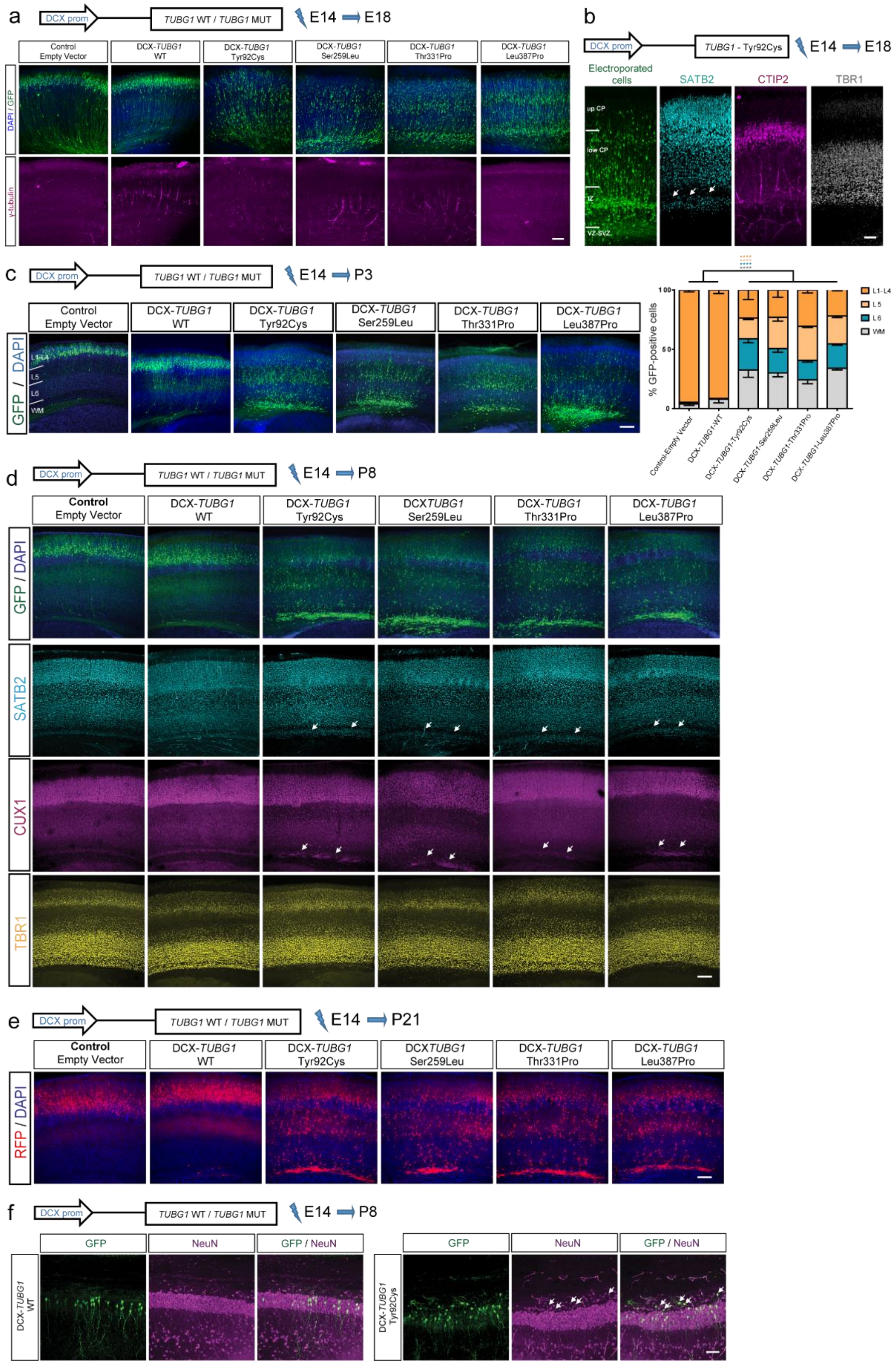

Supplementary Figure 4. Effects of post-mitotic expression of TUBG1 variants on neuronal positioning.

(**a**) Gamma-tubulin staining (magenta) in E18.5 brains electroporated at E14.5 with control-empty vector, the DCX driven WT form or one of the four TUBG1 mutated forms and co-electroporated with a NeuroD driven GFP reporter (green), counterstained with DAPI (blue). Scale bar 150μm. (**b**) E18.5 brains electroporated at E14.5 with DCX-Tyr92Cys and stained with the upper-layer marker SATB2 (cyan), lower layers markers CTIP2 (magenta) and TBR1 (gray). White arrows show $SATB2^+$ cells in the IZ. Scale bar 50μm.(**c**) Left: coronal sections of P3 brains electroporated at E14.5 with control-empty vector, the DCX driven WT form or one of the four TUBG1 mutated forms and co-electroporated with a NeuroD driven GFP reporter (green), counterstained with DAPI (blue). Scale bar 200μm. Right: Percentage fluorescent neurons in the upper cortical layers L2-L4, Layer 5 (L5), Layer 6 (L6) and white matter (WM), Data are from at least 3 pups per condition; Two-way ANOVA with Tukey's multiple comparisons test, **** $p < 0.0001$, compared to control-empty vector and WT-TUBG1. (**d**) P8 brains electroporated at E14.5 with control-empty vector, the DCX driven WT form or one of the four TUBG1 mutated forms and co-electroporated with a NeuroD driven GFP reporter (green). Sections were stained with upper layer markers CUX1 (magenta) or SATB2 (cyan), lower layer marker TBR1 (yellow) and counterstained with DAPI (blue). White arrows show $SATB2^+$ and $CUX1^+$ cells in the WM. Scale bar 250μm. (**e**) P21 brains electroporated at E14.5 with control-empty vector, the DCX driven WT form or one of the four TUBG1 mutated forms and co-electroporated with an RFP reporter (red), counterstained with DAPI (blue). Scale bar 200μm. (**f**) Electroporation of DCX-TUBG1-Y92C or WT together with GFP in the ammonic neuroepithelium at E14.5. Images show coronal sections of the CA1 region of the hippocampus at P8 counterstained with NeuN neuronal marker (magenta). Scale bar 50μm. Arrows indicate ectopic double positive GFP^+NeuN^+ cells. CP, cortical plate; IZ, intermediate zone; VZ/SVZ, ventricular zone/subventricular zone. Data are presented as mean ± s.e.m. Source data are provided in the Source Data file.

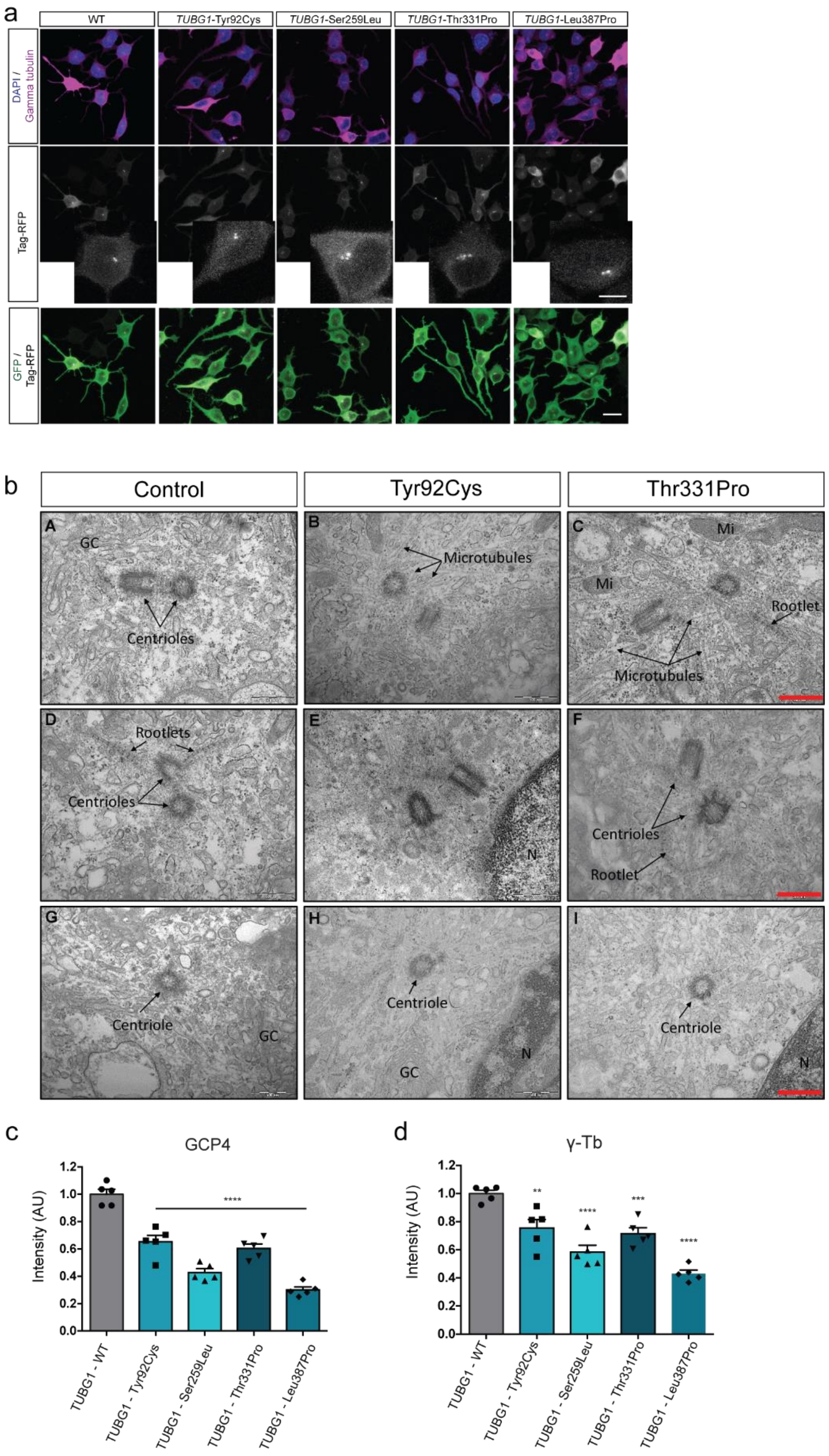

Supplementary Figure 5. Mutant TUBG1 Interactions within gamma-tubulin complexes.

(**a**) Immunofluorescent images of differentiated Neuro2A cells transfected with WT or mutant TUBG1 fused to RFP together with a GFP-expressing reporter construct. Cells were immunolabelled for γ-tubulin (magenta), RFP (grey) and GFP (green) and counterstained with DAPI (blue). Scale bar 20μm; 5 μm for insets. (**b**) Transmitionelectron microscopy from control and patients' derived fibroblasts, showing perpendicular centrioles (A-F), and cross section of centrioles showing the nine triplets microtubules structure (G-I). Scale bars 500nm. An average of 100 cells per condition were analyzed, n=4 independent experiments. GC: Golgi complex, Mi: mitochondria, N: nucleus. (c-d) Neuro2a cells expressing tagRFP-tagged mouse Tubg1 (WT control) or its mutated variants were immunoprecipitated and after blotting probed with antibodies to GCP4 and gamma-tubulin (γ-Tb). Densitometric quantification of immunoblots shown in Figure 4h, stained with anti-GCP4 (**c**) and anti-gamma-tubulin (**d**) antibodies, normalized to WT control and to the amount of tag RFP in individual samples. Data are presented as mean ± s.e.m.; n=5 independent experiments; one-way ANOVA with Bonferroni's multiple comparisons test, ** $p < 0.01$, *** $p < 0.001$, ****$p < 0.0001$. Source data are provided in the Source Data file.

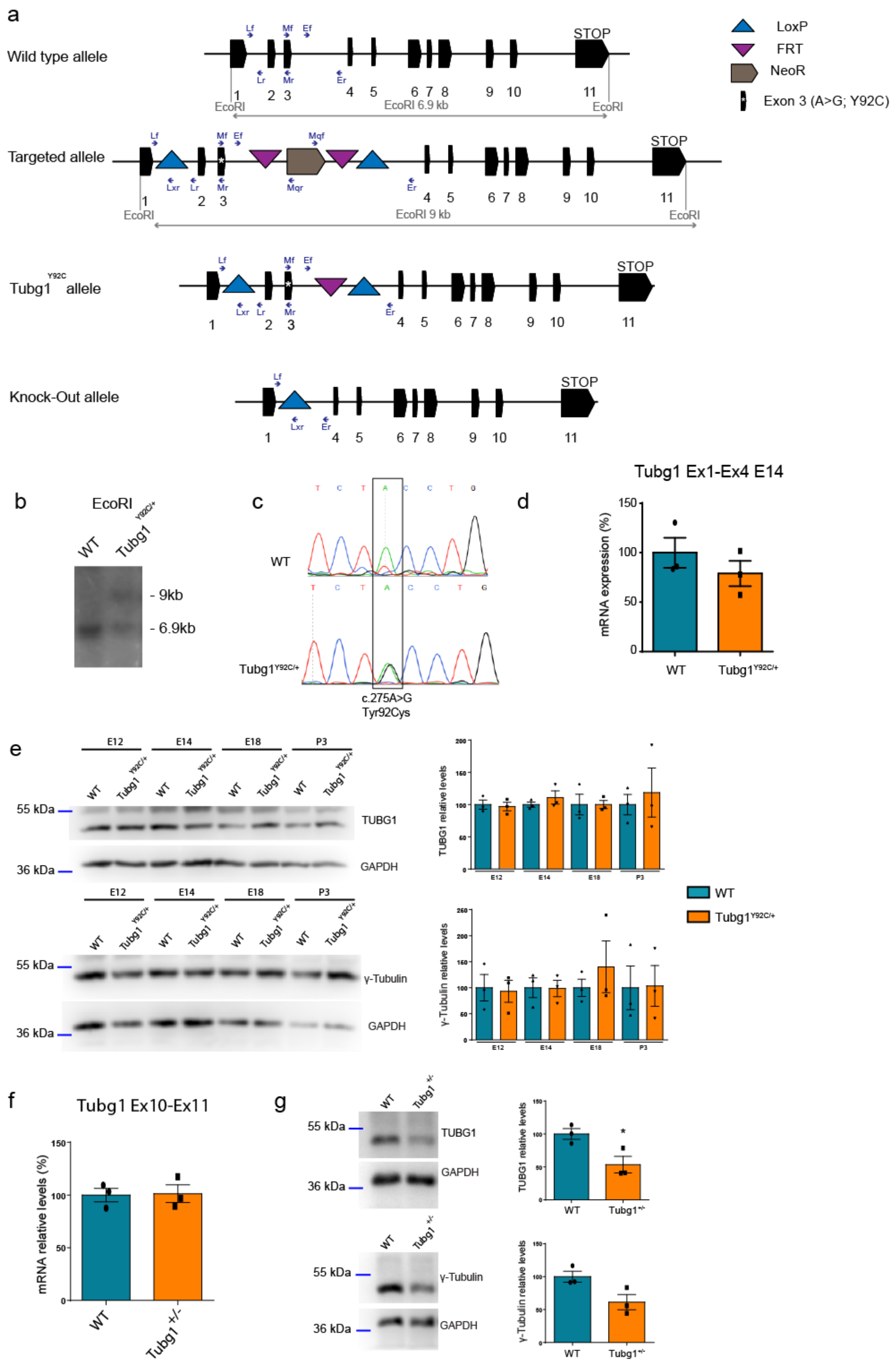

Supplementary Figure 6. Generation of Tubg1$^{Y92C/+}$ knock-in and knock-out mice.

(**a**) Schematic representation of the wild type allele and the targeted vector used for the generation of Tubg1$^{Y92C/+}$ and knock-out alleles. The point mutation is represented by an asterisk in Exon 3. The floxed Neo selection cassette was removed by in vivo breeding with Flp deleter mice to obtain the Tubg1$^{Y92C/+}$ mice. Knock-out mice were obtained by subsequent breeding with a Rosa-Cre deleter line. Position of primers used for genotyping is represented in each allele. The size of EcoRI restriction fragments is shown for the WT and targeted allele. (**b**) DNA from the recombinant selected Tubg1$^{Y92C/+}$ ES cell clone was analyzed by southern blot by restriction with EcoRI and hybridization with a 3'external probe. The WT allele is 6.9kb; the Tubg1$^{Y92C/+}$ allele is 9kb. (**c**) Sequencing results of RT-PCR products using total brain RNA extracted from WT and Tubg1$^{Y92C/+}$ mice with primers located in exons 1 and 4 showing the presence in the KI model of the Tyr92Cys variant at a heterozygous state. (**d**) Tubg1 mRNA expression levels quantification by RT-qPCR using total brain RNA extracted from WT and Tubg1$^{Y92C/+}$ mice at E14 and normalized to actin, showing no effect of the mutation on mRNA levels. WT mRNA expression was considered as 100%; n=3 embryos. (**e**) Left: Protein levels of TUBG1 (upper panel) and total gamma-tubulin (lower panel) in WT and Tubg1$^{Y92C/+}$ mouse brains during development (E12, E14, E18 and P3) detected by immunoblotting. Right: densitometry quantitation of immunoblots showing similar levels in WT and KI mice, n=3 mice. (**f**) Tubg1 mRNA levels quantification by RT-qPCR using total brain RNA extracted from WT and knock-out Tubg1$^{+/-}$ mice at E14, normalized to actin. WT mRNA expression was considered as 100%, n=3 embryos. (**g**) Right: protein levels of TUBG1 (upper panel) and total gamma tubulin (lower panel) in E14 WT and Tubg1$^{+/-}$ mice. Right: densitometry quantification of immunoblots showing reduced TUBG1 protein levels in Tubg1$^{+/-}$ animals, n=3 mice, unpaired two-tailed t-test, * $p < 0.05$. Data are presented as mean ± s.e.m. Source data are provided in the Source Data file.

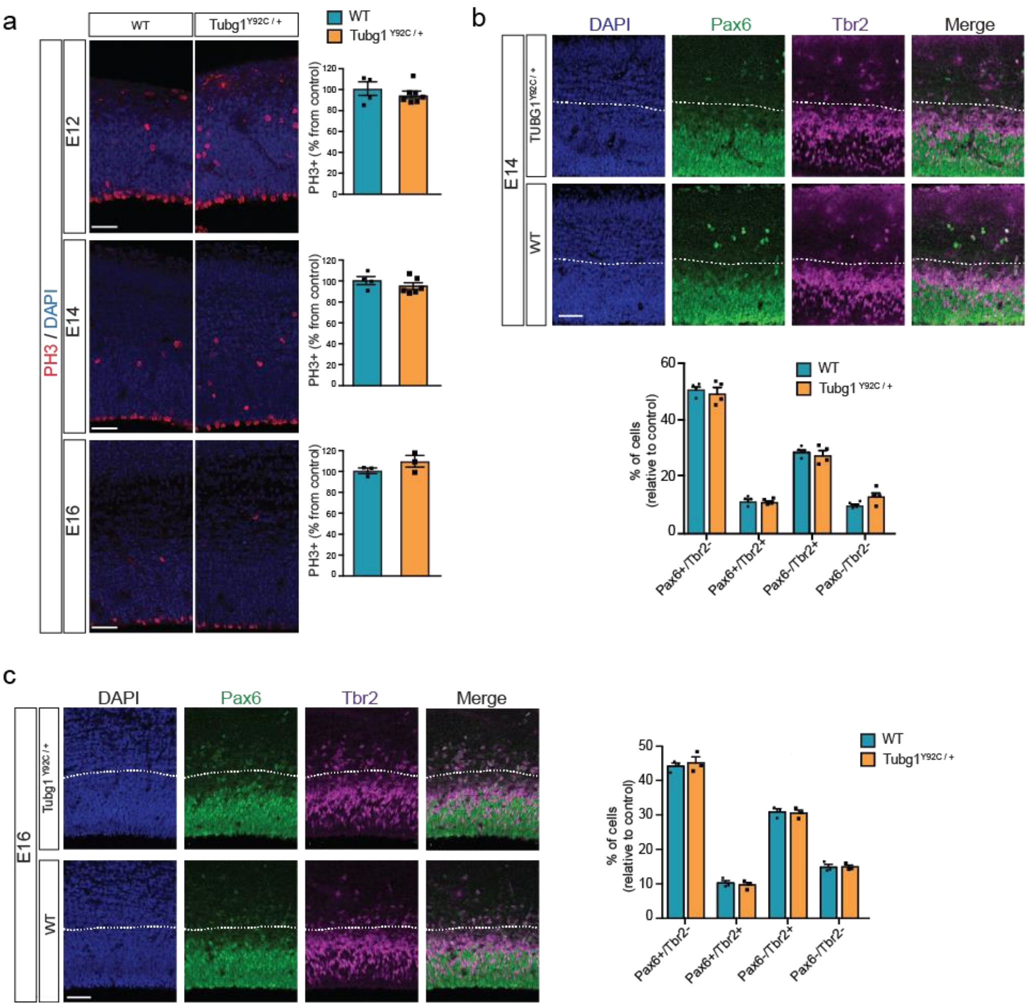

Supplementary Figure 7. Progenitors proliferation in Tubg1$^{Y92C/+}$ knock-in mice. (**a**) Left: coronal sections of E12, E14 and E18 cortices from WT or Tubg1$^{Y92C/+}$ mice immunolabeled for the mitotic marker PH3 (red) and counterstained with DAPI (blue). Scale bar 50µm. Right: PH3^{+} cells in the ventricular zone were counted and shown as a percentage from the control, n≥4 embryos per group, unpaired two-tailed t-test. (**b** and **c**) Left: coronal sections of E14 and E16 cortices from WT or Tubg1$^{Y92C/+}$ mice immunolabeled for the apical progenitor marker Pax6 (green) and the basal progenitor marker Tbr2 (magenta) and counterstained with DAPI (blue). Dotted line delimits the VZ/SVZ for quantifications. Scale bar 50µm. Right: relative percentages of apical progenitors (Pax6^{+}Tbr2^{-}), new-born intermediate progenitors (Pax6^{+}Tbr2^{+}), basal progenitors (Pax6^{-}Tbr2^{+}) and new-born neurons (Pax6^{-}Tbr2^{-}) were calculated and shown as a percentage from control; n≥3 embryos per group, 2-way ANOVA with Sidak's multiple comparison test. Data are presented as mean ± s.e.m. Source data are provided in the Source Data file.

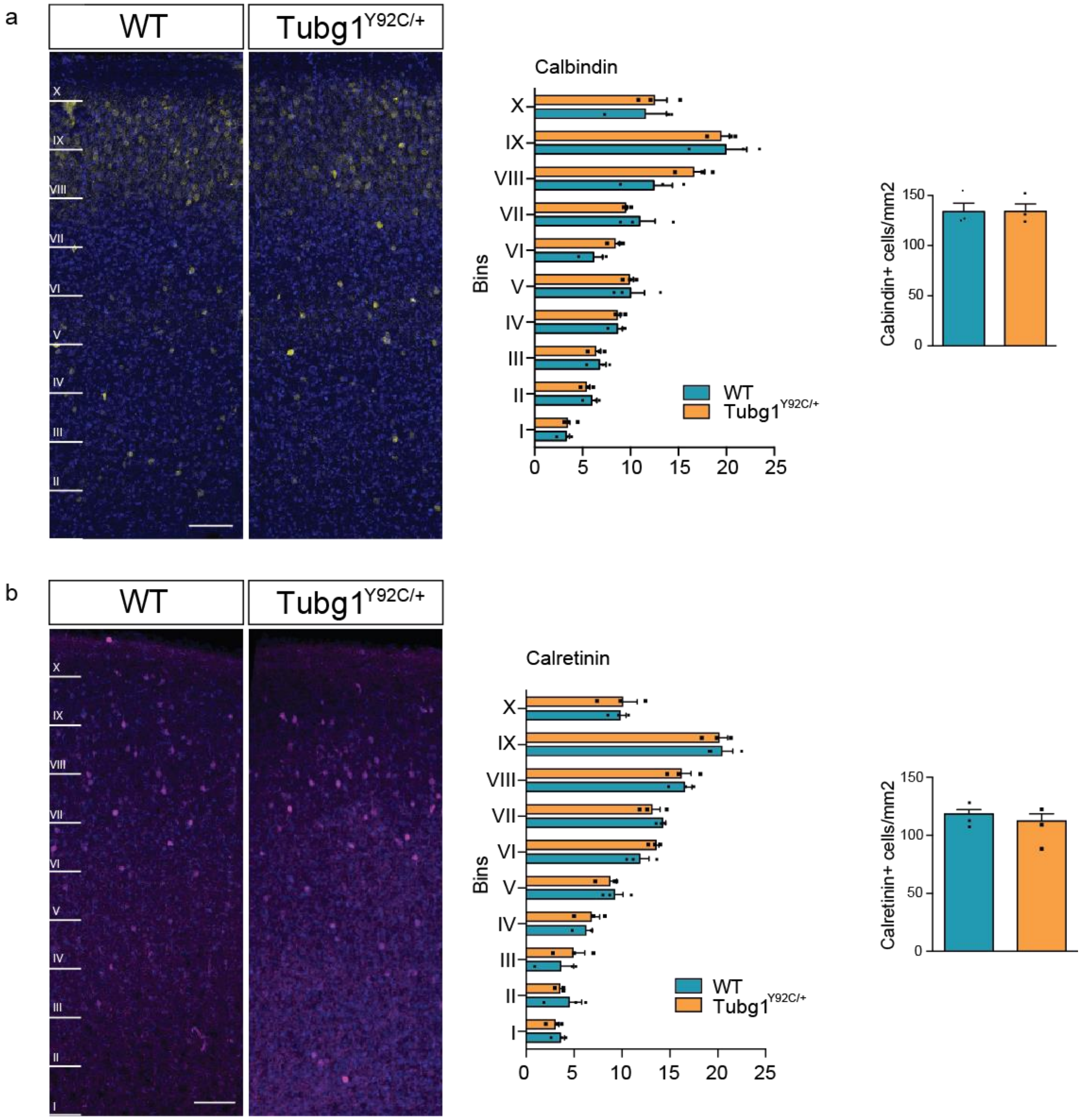

Supplementary figure 8. Interneurons studies in Tubg1$^{Y92C/+}$ mice. Images on the left show coronal sections of 16 weeks-old heterozygous knock-in mice, stained for calbindin^{+} (**a**-yellow) or calretinin^{+} (**b**-magenta) interneurons, counterstained with DAPI (blue). The cortical plate was divided into 10 equal bins. Scale bars 100µm. Histograms on the right represent the distribution of positive cells within the bins in the cortical plate (left panels) and the number of positive cells/mm$_2$ (right panels); n=3 mice per group; two-way ANOVA (left panels) and unpaired two-tailed t-test (right panels). Data are presented as mean ± s.e.m. Source data are provided in the Source Data file.

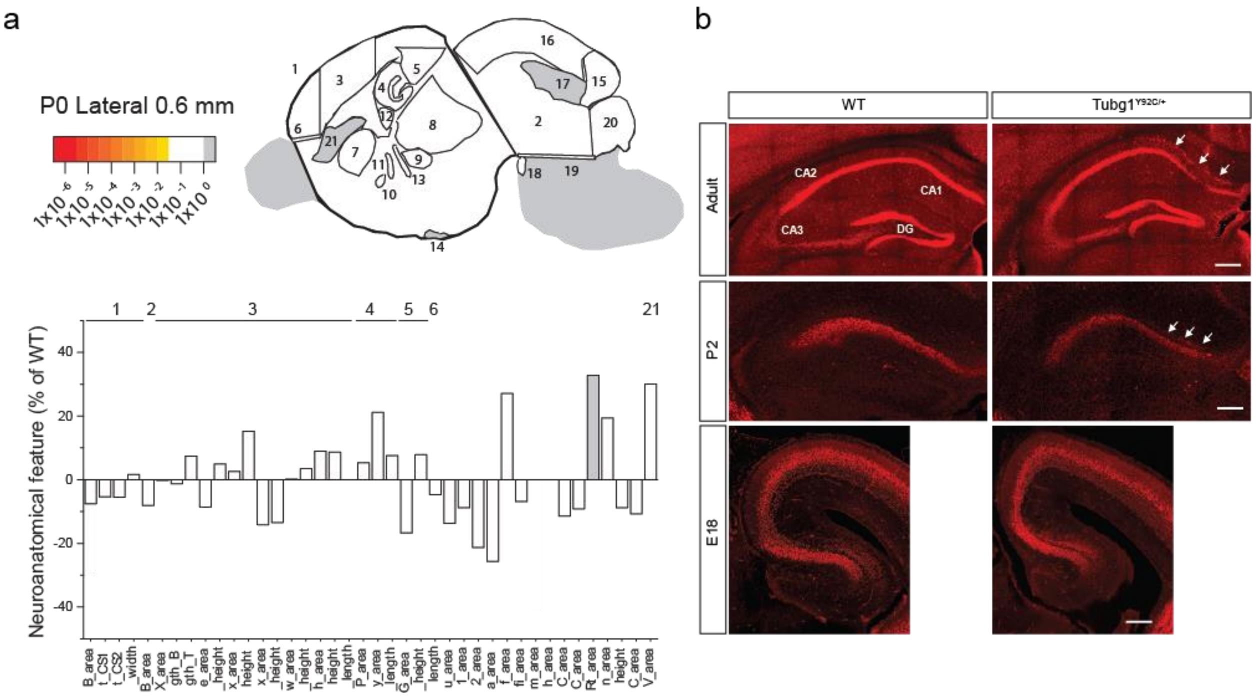

Supplementary figure 9. Neuroanatomical studies in Tubg1$^{Y92C/+}$ mice. (**a**) Upper panel: schematic representation of a brain section at Lateral 0.60 mm at postnatal day 0 (P0) plotted according to p-values. White coloring indicates a p-value higher than 0.05 and grey shows not enough data to calculate a p-value. Lower panel: histogram showing percentage of increase or decrease of parameters in measured areas as compared to matched controls, at Lateral +0.60 mm, n= 5 WT and 4 Tubg1$^{Y92C/+}$ pups, Unpaired two-tailed t-test. Source data are provided in the Source Data file. (**b**) Coronal sections of the hippocampus from control WT and Tubg1$^{Y92C/+}$ mice stained with CTIP2 (red), at adult stage (scale bar 300μm), P8 (scale bar 200 μm) and E18 (scale bar 150 μm). White arrows show ectopic neurons in the stratum oriens of the CA1 region.

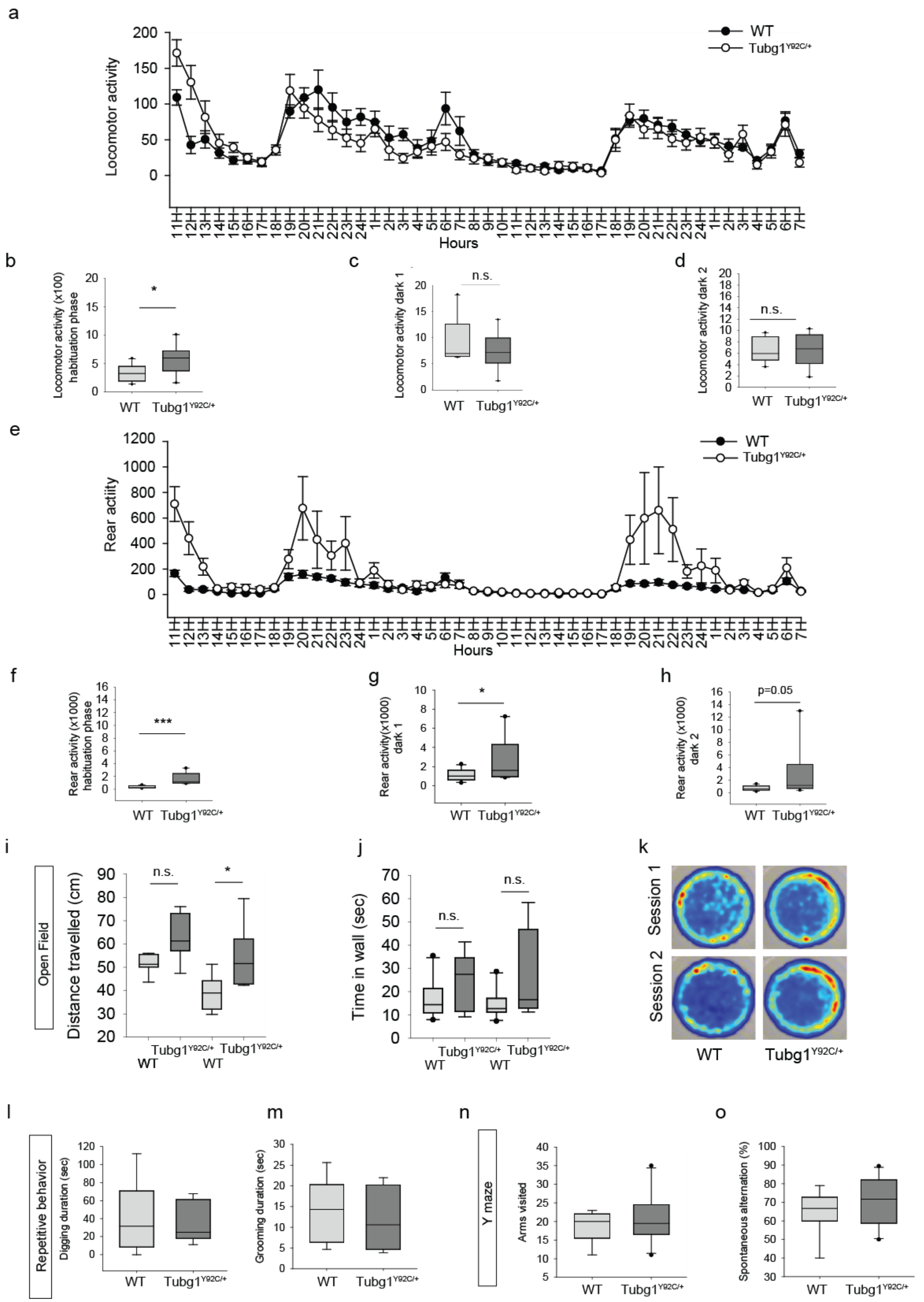

Supplementary figure 10. Behavioral studies in Tubg1$^{Y92C/+}$ mice.

(**a**) Graph shows locomotor activity (count) per hour, during 45-hours of test. Boxplots show locomotor activity (count) during the habituation phase (11:00 to 19:00, **b**), during the first dark phase (19:00 to 7:00, **c**) and finally during the second dark phase (**d**). (**e**) Graph shows rearing behavior (count) per hour, during 45-hours of test. Boxplots show rearing behavior (count) during the habituation phase (11:00 to 19:00, **f**), during the first dark phase (19:00 to 7:00, **g**) and finally during the second dark phase (h). (**i-k**) Open field test. 2 sessions of 14min were done at 24 hours interval. Exploratory locomotor activity is represented by the distance travelled by mice (i) and the time spent near the wall area (**j**). (**k**) Heatmap showing the time spent in the arena during the 14 min of the session 1 and session 2 by genotype. (**l**-**m**) Repetitive behaviors. Digging (**l**) and grooming (**m**) duration in seconds during 10 min of observation in a novel home-cage environment. (**n**-**o**) Continuous spontaneous alternation test. The number of arms visited (**n**) and spontaneous alternation (number of alternations divided by the number of alternation opportunities namely, total arm entries minus one, (**o**) are presented in box plots with center line representing the median, end of boxes 25th and 75th percentile and whiskers 10th and 90th percentiles. Data form at least 9 animals per genotype are presented as mean ± s.e.m and were analyzed with one-way ANOVA with Tukey's post hoc test, * $p < 0.05$, *** $p < 0.001$, ns: not significant vs WT. Source data are provided in the Source Data file.

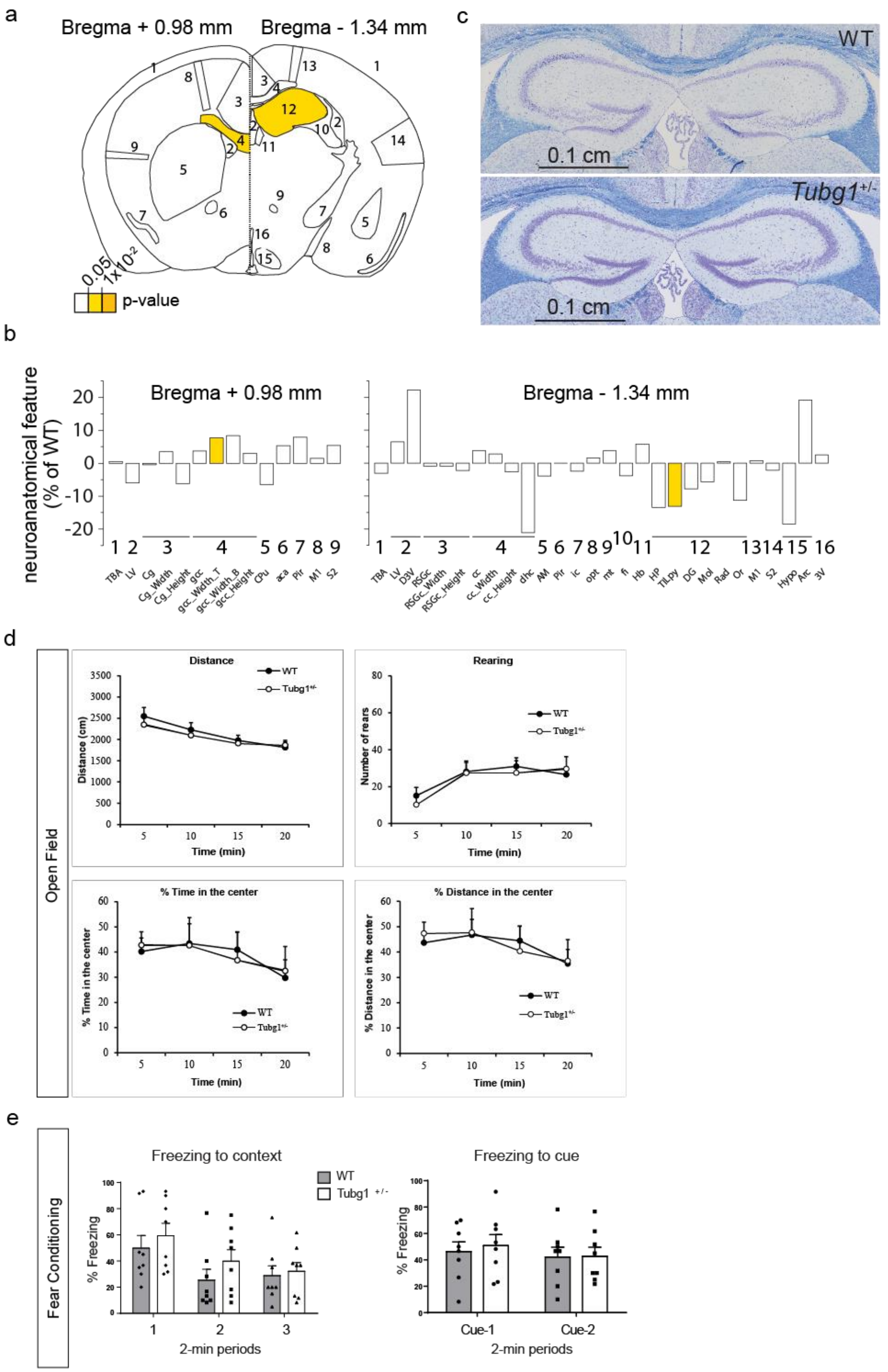

Supplementary figure 11. Neuroanatomy and behavior of Tubg1$^{+/-}$ mice. (**a**) Schematic representation of brain regions in male mice at 16 weeks of age plotted in coronal planes according to p-values. Left: image represents

a section at Bregma +0.98 mm, right: a section at Bregma -1.34 mm. Yellow color indicates p-value below the threshold of 0.05. (**b**) Histograms showing percentage of increase or decrease of parameters in measured areas as compared to the controls, at Bregma +0.98 mm (left panel) and Bregma -1.34 mm (right panel). Numbers indicate studied areas: 1=total brain area, 2=lateral ventricles, 3=cingulate cortex (Bregma +0.98 mm) and retrosplenial cortex (Bregma -1.34 mm), 4=corpus callosum, 5=caudate putamen (Bregma +0.98 mm) and amygdala (Bregma -1.34 mm), 6=anterior commissure (Bregma +0.98 mm) and piriform cortex (Bregma -1.34 mm), 7=piriform cortex (Bregma +0.98 mm) and internal capsule (Bregma -1.34 mm), 8=motor cortex (Bregma +0.98 mm) and optic tract (Bregma -1.34 mm), 9=somatosensory cortex (Bregma +0.98 mm) and mammilo-thalamic tract (Bregma -1.34 mm), 10=fimbria, 11=habenula, 12=hippocampus, 13=primary motor cortex, 14=secondary somatosensory cortex, 15=hypothalamus, and 16=third ventricle. Only two parameters are mildly affected: the width of the genu of the corpus callosum (+7%, P=0.017) and the total internal length of the pyramidal cells of the hippocampus (-13%, P=0.019). Data were analyzed using a two-tailed Student t-test of equal variance, n= 12 mice (n=3 per group, male and female) (**c**) Nissl-luxol double-stained coronal sections of $Tubg1^{+/-}$ mice (bottom panel) and their matched controls (top panel) at Bregma -1.34 mm, showing no cellular heterotopia throughout the section, scale bar 0.1 cm. No differences were observed between male and female mice. (**d**) Open field assay. The distance travelled, the number of rears and percentage of time spent in the center were comparable between WT and $Tubg1^{+/-}$ mice. Data were analyzed using repeated measures ANOVA, n=8 animals per group. (**e**) Fear conditioning assay. Duration of immobility during habituation and percentage of freezing during context and cue sessions were comparable between genotypes. Results were analyzed using repeated measures ANOVA, n=8 animals per group. Data are presented as mean ± s.e.m. Source data are provided in the Source Data file.

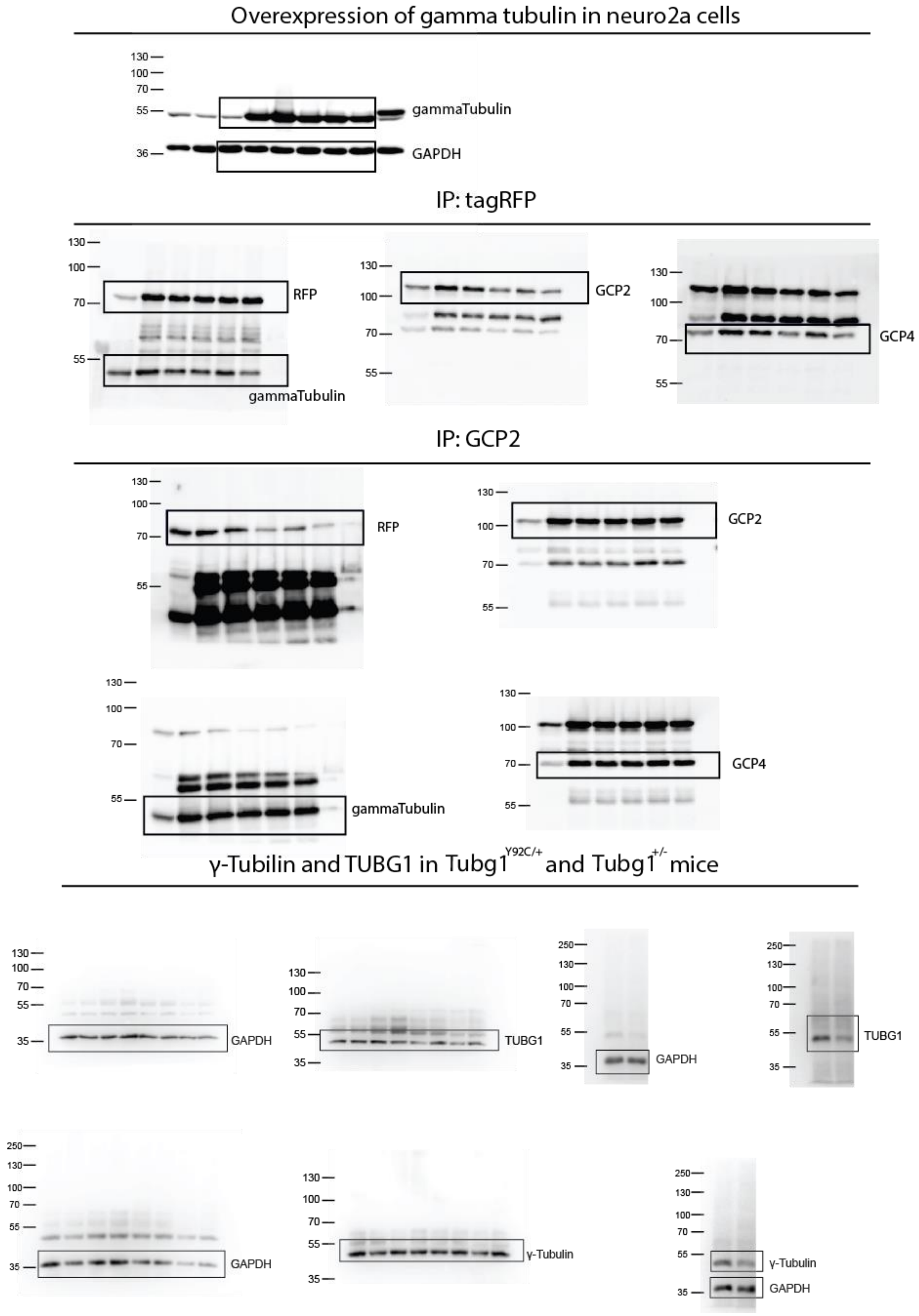

Supplementary figure 12. Most important western blots.
