# Supplementary material for: TUBG1 missense variants underlying cortical malformations disrupt neuronal locomotion and microtubule dynamics but not neurogenesis
Source: Nat Commun. 2019 May 13;10:2129. doi: 10.1038/s41467-019-10081-8 (PMC6513894; doi:10.1038/s41467-019-10081-8)
Supplement: Supplementary file 4 — Description of Additional Supplementary Files [file 41467_2019_10081_MOESM4_ESM.pdf]

Description of Additional Supplementary Files

File Name: Supplementary Data 1
Description: List and description of the 166 neuroanatomical parameters quantified in the morphometric analysis of adult TUBG1 knock-in mice.

File Name: Supplementary Data 2
Description: List and description of the 40 neuroanatomical parameters studied in the morphometric analysis of postnatal day 0 TUBG1 knock-in mice.

File Name: Supplementary Data 3
Description: A detailed description of statistics behind all graphical representation of data.

File Name: Supplementary Movie 1
Description: **Migration of pyramidal neurons electroporated with Control Empty-vector.** Neuronal precursors were electroporated at E14.5 with the DCX control Empty-vector along with a GFP reporter gene under the control of a NeuroD promoter. E16.5 brain slices were cultured for one day, and GFP expressing neurons were then imaged for 10 hours.

File Name: Supplementary Movie 2
Description: **Migration of pyramidal neurons electroporated with *TUBG1*-WT.** Neuronal precursors were electroporated at E14.5 with a *TUBG1*-WT construct under the control of a DCX promoter along with a GFP reporter gene under the control of a NeuroD promoter. E16.5 brain slices were cultured for one day, and GFP expressing neurons were then imaged for 10 hours.

File Name: Supplementary Movie 3
Description: **Migration of pyramidal neurons electroporated with TUBG1-Tyr92Cys.** Neuronal precursors were electroporated at E14.5 with a *TUBG1*-Tyr92Cys construct under the control of a DCX promoter along with a GFP reporter gene under the control of a NeuroD promoter. E16.5 brain slices were cultured for one day, and GFP expressing neurons were then imaged for 10 hours.

File Name: Supplementary Movie 4
Description: **Migration of pyramidal neurons electroporated with TUBG1 Thr331Pro.** Neuronal precursors were electroporated at E14.5 with a *TUBG1*-Thr331Pro construct under the control of a DCX promoter along with a GFP reporter gene under the control of a NeuroD promoter. E16.5 brain slices were cultured for one day, and GFP expressing neurons were then imaged for 10 hours.

File Name: Supplementary Movie 5
Description: **Microtubules dynamics in Control HeLa cells:** Representative time-lapse movie of HeLa cells transfected with EB3-GFP and the corresponding control Empty-vector. Images were collected every 500ms for 2 min.

File Name: Supplementary Movie 6
Description: **Microtubules dynamics in *TUBG1*-WT HeLa cells.** Representative time-lapse movie of HeLa cells transfected with EB3-GFP and co-transfected with *TUBG1*-WT. Images were collected every 500ms for 2 min.

File Name: Supplementary Movie 7
Description: **Microtubules dynamics in *TUBG1* Tyr92Cys HeLa cells.** Representative timelapse movie of HeLa cells transfected with EB3-GFP and co-transfected with TUBG1-Tyr92Cys. Images were collected every 500ms for 2 min.

File Name: Supplementary Movie 8
Description: **Microtubules dynamics in *TUBG1* Ser259Leu HeLa cells.** Representative time-lapse movie of HeLa cells transfected with EB3-GFP and co-transfected with *TUBG1*-Ser25.9Leu. Images were collected every 500ms for 2 min.

File Name: Supplementary Movie 9
Description: **Microtubules dynamics in *TUBG1* Thr331Pro HeLa cells.** Representative time-lapse movie of HeLa cells transfected with EB3-GFP and co-transfected with *TUBG1*-Thr331Pro Images were collected every 500ms for 2 min.

File Name: Supplementary Movie 10
Description: **Microtubules dynamics in *TUBG1* Leu387Pro HeLa cells.** Representative time -lapse movie of HeLa cells transfected with EB3-GFP and co-transfected with *TUBG1*-Leu387Pro. Images were collected every 500ms for 2 min.

File Name: Supplementary Movie 11
Description: **Microtubules dynamics in Control 1 fibroblasts.** Representative time -lapse movie of Control-1 fibroblasts transfected with EB3-GFP. Images were collected every 500ms for 2 min.

File Name: Supplementary Movie 12
Description: **Microtubules dynamics in Control 2 fibroblasts.** Representative time -lapse movie of Control-2 fibroblasts transfected with EB3-GFP. Images were collected every 500ms for 2 min.

File Name: Supplementary Movie 13
Description: **Microtubules dynamics in Control 3 fibroblasts.** Representative time -lapse movie of Control-3 fibroblasts transfected with EB3-GFP. Images were collected every 500ms for 2 min.

File Name: Supplementary Movie 14
Description: **Microtubules dynamics in subject-derived TUBG1-Tyr92Cys fibroblasts.** Representative time -lapse movie of subject-derived fibroblasts carrying the Tyr92Cys mutation and transfected with EB3-GFP. Images were collected every 500ms for 2 min.

File Name: Supplementary Movie 15
Description: **Microtubules dynamics in subject-derived TUBG1-Thr331Pro fibroblasts.** Representative time -lapse movie of subject-derived fibroblasts carrying the Thr331Pro mutation and transfected with EB3-GFP. Images were collected every 500ms for 1 min.

File Name: Supplementary Movie 16
Description: **Migration of pyramidal neurons in *Tubg1* WT mice.** Neuronal precursors of *Tubg1* WT mice were electroporated at E14.5 with a GFP reporter gene under the control of a NeuroD promoter. E16.5 brain slices were cultured for one day, and GFP expressing neurons were then imaged for 10 hours.

File Name: Supplementary Movie 17
Description: **Migration of pyramidal neurons in *Tubg1*$^{Y92C/+}$ mice.** Neuronal precursors of *Tubg1*$^{Y92C/+}$ mice were electroporated at E14.5 with a GFP reporter gene under the control of a NeuroD promoter. E16.5 brain slices were cultured for one day, and GFP expressing neurons were then imaged for 10 hours.
